# Disease-specific quality of life after thoracoscopic repair of esophageal atresia: a single-centre cross-sectional study

**DOI:** 10.3389/fped.2026.1844923

**Published:** 2026-07-01

**Authors:** Marcin Łosin, Weronika Lotkowska, Oliwer Sowulewski, Maciej Zagierski, Agnieszka Szlagatys-Sidorkiewicz, Piotr Czauderna

**Affiliations:** 1 Department of Surgery and Urology for Children and Adolescents, Medical University of Gdańsk, Gdańsk, Poland; 2 Department of Paediatrics, Gastroenterology, Allergology & Paediatric Nutrition, Medical University of Gdańsk, Gdańsk, Poland

**Keywords:** EA-QOL questionnaire, eating difficulties, esophageal atresia (EA), quality of life, thoracoscopy (VATS)

## Abstract

**Background:**

Survival after esophageal atresia (EA) repair now exceeds 90%, yet little is known about long-term quality of life in patients treated with thoracoscopy as the primary intended approach. Most published cohorts mix open and thoracoscopic approaches, making it difficult to disentangle disease-related impairment from approach-related morbidity. We set out to describe disease-specific health-related quality of life (HRQoL) in a cohort managed by thoracoscopy as the primary intended approach, compare our data with European reference values, and explore child–parent agreement.

**Methods:**

Cross-sectional survey of 25 respondents (response rate 56%) from 51 children who underwent EA repair with thoracoscopy as the intended primary approach [7 (14%) required intraoperative conversion to open thoracotomy and were retained under an intention-to-treat principle] at a single Polish centre (2006–2025). The validated Polish EA-QOL questionnaire was administered: parent proxy for children aged 2–7 years (*n* = 12), child self-report and parent proxy for those aged 8–17 years (*n* = 13 and *n* = 12, respectively). Agreement between child and parent reports was evaluated with Pearson and Spearman correlations and Bland–Altman analysis. Scores were compared with published Swedish–German reference data, and clinical associations were examined using non-parametric methods.

**Results:**

Overall QoL% ranged from 76% to 79% across age groups. Eating difficulties were the most impaired domain in every questionnaire version (QoL%: 63%–70%), driven largely by problems with food consistency. No domain score differed from reference data after Bonferroni correction, though the Body and Scar domain in older children showed a borderline signal (*p* = 0.049, Cohen's *d* = 0.56). Child–parent agreement was strong (Pearson *r* = 0.83, Bland–Altman bias 0.000, 95% limits of agreement: −0.62 to +0.62). None of the clinical variables tested were associated with HRQoL in bivariate analyses, though the study was not powered for such comparisons.

**Conclusions:**

In this single-centre cohort managed by thoracoscopy as the primary intended approach, disease-specific HRQoL was generally preserved, with eating-related difficulties as the dominant area of residual impairment. These findings add to the limited literature on approach-specific outcomes in EA and support routine long-term monitoring of food texture tolerance and reflux burden.

## 1 Introduction

Esophageal atresia with or without tracheoesophageal fistula (EA/TEF) occurs in roughly 1 in 2,500–3,500 live births (1, 2). Survival has improved to the point where attention has shifted from keeping these children alive to understanding how they actually fare in everyday life (3).

Thoracoscopic repair, introduced by Rothenberg in 2000, has gained ground as an alternative to open thoracotomy (4). Its potential advantages—reduced chest wall trauma, less musculoskeletal morbidity, smaller scars—are appealing, but most studies examining long-term quality of life in EA have enrolled mixed cohorts without stratifying by operative technique (3, 5–8). This matters because thoracotomy carries its own late problems—rib cage asymmetry, scoliosis, winged scapula—that may independently affect body image and physical function (6). Lumping both approaches together makes it harder to determine which problems are intrinsic to EA and which are secondary to the operative route.

Generic instruments such as PedsQL, while widely used, lack the sensitivity to capture the swallowing difficulties, food texture restrictions, and treatment burden that define the daily experience of many EA patients. The EA-QOL questionnaire, developed by Dellenmark-Blom et al. in Sweden and Germany (9), is the only disease-specific measure currently available for this population. It has since been adapted into Polish (10) and more than a dozen other languages (11, 12), but published data on cohorts managed by thoracoscopy as the primary intended approach remain scarce.

In Poland, Rozensztrauch et al. (13) reported generally preserved HRQoL in a mixed Wrocław cohort (*n* = 73) using PedsQL 4.0, with no significant clinical predictors. However, no Polish study has yet applied a disease-specific instrument to a cohort managed by thoracoscopy as the primary intended approach, leaving a gap in the literature both nationally and internationally. Our centre introduced thoracoscopic EA repair in 2006 and has used it as the primary approach since. The present study had three aims: to describe disease-specific HRQoL in this homogeneous cohort using the Polish EA-QOL, to compare our results with published European reference data, and to examine child–parent agreement.

## 2 Materials and methods

### 2.1 Study design and participants

We conducted a cross-sectional survey at the Department of Paediatric Surgery and Urology, Medical University of Gdańsk. All 51 children who underwent surgery with thoracoscopy as the intended primary approach (i.e., thoracoscopic EA repair) between January 2006 and December 2025 constituted the eligible cohort. In keeping with an intention-to-treat principle, the seven patients (14%) who required intraoperative conversion to open thoracotomy were retained within the cohort rather than excluded or analysed as a separate surgical group, since the decision to operate thoracoscopically—and the perioperative course leading to conversion—is itself part of the treatment pathway being evaluated. Six were excluded from the survey: two died perioperatively, and four operated in 2025 had not yet reached the minimum eligible age of 2 years. Of the 45 questionnaire packages distributed, 25 complete responses were returned (56% response rate). The institutional Ethics Committee approved the study; written informed consent or assent was obtained from all participants.

### 2.2 EA-QOL questionnaire

The validated Polish EA-QOL was administered in its three versions (10). For children aged 2–7 years, only the 17-item parent proxy was used. For those aged 8–17, both the 24-item child self-report and the parent proxy were completed. Each item is scored 0–4 (0 = never, 4 = always; higher scores indicate greater impairment). Domain scores were calculated for Eating, Health and Treatment, Other People, and Body and Scar (all versions), plus General Health (8–17 years only). A QoL% transformation was applied as (4 − mean)/4 × 100, so that higher percentages indicate better quality of life.

### 2.3 Clinical data

Medical records were reviewed for: sex, birth weight, prematurity, gestational age, Gross classification, associated anomalies, conversion to open thoracotomy, postoperative complications graded by Clavien–Dindo classification, anastomotic stricture, number of endoscopic dilatations, and gastroesophageal reflux (GER) as documented by the treating clinicians. GER status was also recorded in the questionnaire database for each respondent (yes/no), completed by the parent or treating team at the time of questionnaire administration. In one patient, the parent proxy indicated GER present while the child self-report did not; the parent report was used as the primary GER status for that patient. Because retrospective operative records were expected to under-capture reflux, a dedicated symptom-based reflux questionnaire was additionally distributed to families alongside the EA-QOL packages and completed for 24 of the 25 respondents. This instrument recorded whether GER had been clinically diagnosed, whether the diagnosis had been confirmed endoscopically, the presence of individual reflux-related symptoms (e.g., regurgitation/vomiting, Sandifer posturing, heartburn, recurrent respiratory infections, chronic cough), and the reflux management received—both pharmacological (e.g., proton-pump inhibitors) and surgical (anti-reflux procedure, with type and date). Standardised objective testing (pH-impedance monitoring) and a formally validated reflux questionnaire were not employed; the symptom inventory used here was developed in-house for this study.

### 2.4 Statistical analysis

Total EA-QOL score was the primary outcome; domain-level and clinical correlate analyses were secondary and exploratory. Data are reported as mean ± SD or median [IQR]. Child–parent agreement was assessed with Pearson and Spearman correlations and Bland–Altman analysis (14) (95% limits of agreement = bias ± 1.96 × SD). Between-group comparisons used Mann–Whitney *U* (binary variables) and Spearman correlation (continuous variables). Comparison with Swedish–German reference data (9) used Welch's *t*-test; significance was set at *p* < 0.05. Bonferroni correction was applied where multiple comparisons were performed. Effect sizes (Cohen's *d*) are reported alongside *p*-values throughout. No *a priori* power calculation was performed, and no multivariable model was fitted given the sample size. All analyses were carried out in Python 3.12 (scipy 1.14); we chose not to apply a formal correction for multiple testing beyond the Bonferroni adjustment in the normative comparison, given the small sample and exploratory intent, though we recognise this increases the risk of type I error in the correlate analyses. The results should accordingly be treated as hypothesis-generating. This study is reported in accordance with the STROBE guidelines for cross-sectional observational studies; a completed checklist is provided as Supplementary File 1.

## 3 Results

### 3.1 Cohort characteristics

Table 1 summarises operative and clinical characteristics. Among the 51 children, males predominated slightly (28/51, 55%). Mean birth weight was 3,023 ± 561 g, nine patients (18%) had low birth weight, and all had Gross type C EA with distal TEF. Associated anomalies were present in 34 patients (67%), most commonly cardiac defects (9/51, 18%), consistent with the VACTERL spectrum. Ten patients (20%) had anomalies spanning more than one organ system.

**Table 1:** Operative and clinical characteristics of the study cohort (2006–2025; *N* = 51) and HRQoL survey respondents (*n* = 25).

Characteristic	Total cohort (*N* = 51)	Respondents (*n* = 25)
Sex: Male, *n* (%)	28 (55)	14 (56)
Sex: Female, *n* (%)	23 (45)	11 (44)
Birth weight, mean ± SD (g)	3,023 ± 561	3,159 ± 437
Birth weight, median [IQR] (g)	3,100 [2,900–3,425]	3,300 [2,900–3,500]
Low birth weight (<2,500 g), *n* (%)	9 (18)	2 (8)
Age at surgery, median [IQR] (days)	4 [2–4]	4 [2–4]
Gross type C (EA + distal TEF), *n* (%)	51 (100)	25 (100)
Any associated anomaly, *n* (%)	34 (67)	14 (56)
Cardiac	9 (18)	6 (24)
Growth/endocrine/metabolic/neurological	4 (8)	3 (12)
Musculoskeletal/limb	3 (6)	2 (8)
Vascular/other	3 (6)	3 (12)
Syndromic/genetic (incl. trisomy 21)	3 (6)	2 (8)
Renal/urinary tract	1 (2)	2 (8)
Gastrointestinal/anorectal	1 (2)	1 (4)
Conversion to open thoracotomy, *n* (%)	7 (14)	3 (13)
Operative duration, median [IQR] (min)	150 [120–199]	—
ICU stay, median [IQR] (days)	9 [7–12]	—
Hospital discharge age, median [IQR] (days)	24.5 [19–31]	—
Clavien–Dindo Grade I	25 (49)	—
Clavien–Dindo Grade II	14 (27)	—
Clavien–Dindo Grade III	4 (8)	—
Clavien–Dindo Grade IV	6 (12)	—
Clavien–Dindo Grade V (death)	2 (4)	—
Clavien–Dindo Grade ≥ III (major)	12 (24)	—
Anastomotic (leak/dehiscence)	10 (20)	—
Respiratory	13 (25)	—
Infectious	5 (10)	—
Stricture requiring dilatation, *n* (%)	31 (61)	17 (68)
Dilatations, median [IQR] (all patients)	1 [0–3]	2 [0–3]
Dilatations, median [IQR] (dilated only)	2 [1–4], max 10	2 [2–4]
GER formally documented, *n* (%)	13 (25)	—
Age at HRQoL assessment, median [IQR] (y)	—	8 [4–12]
EA-QOL: 2–7 years parent proxy	—	*n* = 12
EA-QOL: 8–17 years child self-report	—	*n* = 13
EA-QOL: 8–17 years parent proxy	—	*n* = 12

EA, esophageal atresia; TEF, tracheoesophageal fistula; ICU, intensive care unit; GER, gastroesophageal reflux; IQR, interquartile range. Associated anomaly and complication categories are not mutually exclusive.

All procedures were performed within the first week of life [median day 4 (IQR: 2–4)]. Conversion to open thoracotomy was needed in 7 cases (14%), most often because of inadequate pouch mobilisation or poor operative conditions (4 cases) and intraoperative complications precluding safe completion thoracoscopically—azygos vein injury with bleeding (2 cases) and proximal oesophageal perforation (1 case). Median ICU stay was 9 days [IQR: 7–12]; median hospital discharge occurred at 24.5 days of age [IQR: 19–31].

Two patients died (Clavien–Dindo Grade V, 3.9%): one from anastomotic leak complicated by haemodynamic collapse, the other from respiratory failure in a premature neonate with intrauterine growth restriction. Major complications (Grade ≥ III) occurred in 12/51 (24%). Anastomotic leak or dehiscence was the most frequent serious problem (10/51, 20%); respiratory complications affected 13/51 (25%).

Anastomotic stricture requiring dilatation developed in 31/51 patients (61%). Among those dilated, the median number of sessions was 2 [IQR: 1–4], maximum 10. Strictures were managed by endoscopic balloon dilatation; in the two most refractory cases this was supplemented by surgical stricture resection with redo anastomosis via thoracotomy. Oesophageal stents were not used in this anastomotic-stricture population; in our practice stenting is reserved for the complicated management of long-gap oesophageal discontinuity, which none of these Gross type C patients had. GER was documented in clinical records for 13/51 patients (25%). However, in the questionnaire database—where GER status was available for 24 of 25 respondents—13/24 (54%) reported reflux. In the dedicated reflux questionnaire, of these 13 children with reported reflux, 10 had endoscopically confirmed disease; 12 were receiving pharmacological treatment (predominantly proton-pump inhibitors, with one child on a combination of an alginate preparation and a proton-pump inhibitor); and one child had undergone an anti-reflux operation (Nissen fundoplication, performed in 2016). This discrepancy likely reflects incomplete retrospective documentation rather than true absence of reflux, and it is worth keeping in mind when interpreting the Eating domain.

### 3.2 HRQoL scores by age group and domain

Table 2 shows EA-QOL domain scores. Overall total scores were 0.94 ± 0.44 (2–7 year proxy), 0.87 ± 0.53 (8–17 year child self-report), and 0.87 ± 0.46 (8–17 year parent proxy), corresponding to QoL% of 76%, 78%, and 78%, respectively. Eating was the most impaired domain across all three versions, with mean scores of 1.21, 1.48, and 1.42 (QoL% 70%, 63%, 65%). At the other end, social functioning (Other People) was best preserved—particularly in the 8–17 year self-report group, where the mean was 0.30 (QoL% 93%). The gap between the worst and best domains amounted to 20–30 percentage points.

**Table 2:** EA-QOL domain scores by questionnaire version.

Domain	2–7 years proxy (*n* = 12)	QoL%	8–17 years child SR (*n* = 13)	QoL%	8–17 years parent (*n* = 12)	QoL%
Total score	0.94 ± 0.44	76.4	0.87 ± 0.53	78.2	0.87 ± 0.46	78.2
Eating	1.21 ± 0.45	69.7	1.48 ± 0.99	63.1	1.42 ± 0.81	64.6
Health and Treatment	1.03 ± 0.92	74.2	1.00 ± 0.72	75.0	0.94 ± 0.58	76.4
Other People	0.83 ± 0.49	79.2	0.30 ± 0.64	92.6	0.50 ± 0.62	87.5
Body and Scar	0.50 ± 0.60	87.5	0.72 ± 0.45	82.1	0.75 ± 0.45	81.2
General Health	—	—	0.87 ± 0.83	78.2	0.67 ± 0.74	83.3
Most impaired item	P04: 3.67	8.3	P04: 2.92	26.9	P04: 2.92	27.1
Least impaired item	P03: 0.17	95.8	P14: 0.15	96.2	P19: 0.17	95.8

Scale: 0 (never) to 4 (always); higher = greater impairment. QoL% = (4 − mean)/4 × 100; higher = better quality of life. SR, self-report. P04, difficulty eating food of specific consistency; P03, food refusal distressing others; P14, body image distress; P19, embarrassment about body/scar.

### 3.3 Item-level analysis

Item P04, which asks about difficulty eating food of a specific consistency (e.g., solid or lumpy food), was the single most affected item in every questionnaire version: mean 3.67 ± 0.89 (2–7 year proxy), 2.92 ± 1.50 (8–17 year child), and 2.92 ± 1.44 (8–17 year parent). The second most impaired items were P07 (food sticking in the throat, 8–17 year child self-report, mean 1.69) and P21 (concerns about body or scar, 8–17 year parent proxy, mean 1.75). The least affected items were P03 (food refusal distressing others, mean 0.17 in the 2–7 year group) and P14 (body image distress, mean 0.15 in the 8–17 year child report).

### 3.4 Comparison with reference data

Table 3 presents scores alongside published Swedish–German reference values (9). Only the Body and Scar domain in the 8–17 year child self-report showed a nominally significant difference (0.72 vs. 0.42, *p* = 0.049, Cohen's *d* = 0.56), but this did not survive Bonferroni correction for five simultaneous comparisons (adjusted threshold *p* < 0.01). All other domains and total scores were statistically indistinguishable from the norms (all *p* > 0.05). Reference values for Health & Treatment and General Health in the 8–17 year parent proxy were not reported in the original publication and could therefore not be compared.

**Table 3:** Comparison with Swedish–German reference data (9).

Domain	Gdańsk (mean ± SD)	Norms (mean ± SD; *n*)	*p*-value	Cohen's *d*
2–7 years—parent proxy	*n* = 12	*n* = 53		
Total	0.944 ± 0.439	0.860 ± 0.500	0.567	+0.17
Eating	1.213 ± 0.449	1.240 ± 0.720	0.870	−0.04
Health and treatment	1.033 ± 0.922	0.680 ± 0.650	0.230	+0.50
Other people	0.833 ± 0.492	0.630 ± 0.600	0.231	+0.35
Body and scar	0.500 ± 0.601	0.460 ± 0.660	0.840	+0.06
8–17 years—child SR	*n* = 13	*n* = 62		
Total	0.871 ± 0.532	0.720 ± 0.440	0.353	+0.33
Eating	1.477 ± 0.988	1.000 ± 0.800	0.123	+0.57
Health and treatment	1.000 ± 0.719	0.640 ± 0.650	0.114	+0.54
Other people	0.295 ± 0.635	0.560 ± 0.670	0.192	−0.40
Body and scar	0.718 ± 0.448	0.420 ± 0.550	0.049*	+0.56
8–17 years—parent proxy	*n* = 12	*n* = 71		
Total	0.873 ± 0.464	0.690 ± 0.430	0.222	+0.42
Eating	1.417 ± 0.811	1.020 ± 0.780	0.136	+0.51
Other people	0.500 ± 0.615	0.470 ± 0.570	0.877	+0.05
Body and scar	0.750 ± 0.452	0.460 ± 0.530	0.062	+0.56

Scale: 0–4 (lower = better). Cohen's *d*: positive = greater impairment in Gdańsk cohort.

* Non-significant after Bonferroni correction (adjusted threshold *p* < 0.01). Health and Treatment and General Health norms were not reported for the 8–17 years parent proxy version.

### 3.5 Child–parent agreement

**Figure 1:**
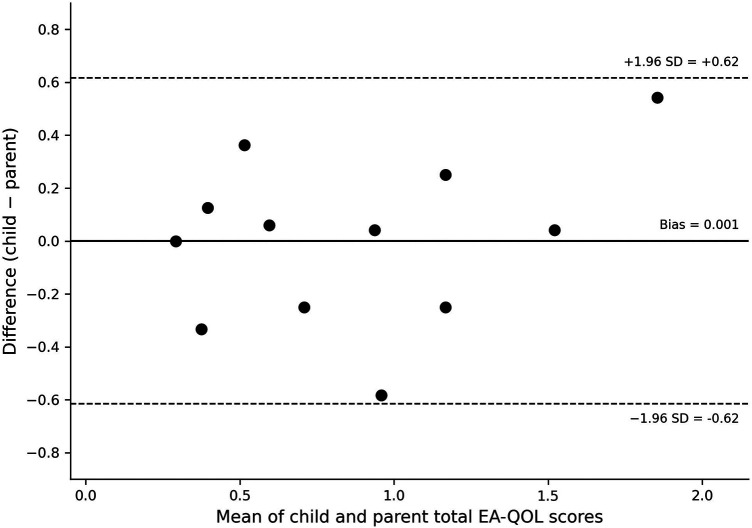
Bland–Altman plot of child self-report versus parent proxy total EA-QOL scores in children aged 8–17 years (*n* = 12 matched pairs). *X*-axis: mean of child and parent scores. *Y*-axis: difference (child minus parent). Solid line: mean bias (0.000). Dashed lines: 95% limits of agreement (+0.62 and −0.62). Data points scatter symmetrically around zero, indicating strong agreement with no systematic directional bias.

Twelve matched child–parent pairs were available (one child self-report lacked a corresponding parent form). Table 4 and Figure 1 present the Bland–Altman analysis. The correlation was strong: Pearson *r* = 0.83 (*p* = 0.001), Spearman *ρ* = 0.83 (*p* = 0.001). The mean difference (bias) was 0.000 (95% CI: −0.20 to +0.20), indicating no systematic tendency for either informant to report higher impairment. The 95% limits of agreement ranged from −0.62 to +0.62. A paired *t*-test confirmed the absence of systematic bias (*t* = 0.005, *p* = 0.996).

**Table 4:** Bland–Altman analysis of child self-report vs. parent proxy total EA-QOL scores, aged 8–17 years (*n* = 12 matched pairs).

Parameter	Value
*n* matched pairs	12
Child self-report, mean ± SD	0.874 ± 0.556
Parent proxy, mean ± SD	0.873 ± 0.464
Mean difference (child–parent) = Bias	0.000
95% CI of bias	[−0.200, +0.200]
SD of differences	0.314
95% Limits of agreement	[−0.615, +0.616]
Pearson *r* (p)	0.825 (*p* = 0.001)
Spearman *ρ* (p)	0.827 (*p* = 0.001)
Paired *t*-test (p)	*t* = 0.005, *p* = 0.996

Positive bias indicates child rates greater impairment than parent. Limits of agreement = bias ± 1.96 × SD. See Figure 1.

### 3.6 Clinical correlates of HRQoL

None of the clinical variables examined showed a statistically significant bivariate association with total EA-QOL score: postoperative complications (*p* = 0.20), stricture (*p* = 0.91), conversion (*p* = 1.00), associated anomalies (*p* = 0.72), sex (*p* = 0.57), age at assessment (*ρ* = −0.06, *p* = 0.77), birth weight (*ρ* = 0.03, *p* = 0.93), number of dilatations (*ρ* = −0.16, *p* = 0.46), and GER status (*p* = 0.750, Cohen's *d* = −0.04). The pattern is consistent with findings from other small EA cohorts (13, 15), though it is important to recognise that with 25 respondents and no multivariable adjustment, we lacked the power to detect anything but large effects. Absence of statistical significance here should not be mistaken for evidence of absent effects.

Because seven patients required conversion to open thoracotomy, we examined the converted patients separately. Three converted children were among the survey respondents (one in the 2–7-year proxy version and two in the 8–17-year group, one of whom also had a matched parent proxy). Their total EA-QOL scores (mean: 0.89, range: 0.58–1.29; QoL%: ≈78%) were essentially indistinguishable from those of the respondents whose repair was completed thoracoscopically (mean: 0.90; QoL%: ≈78%; Mann–Whitney *p* = 0.88). With only three converted respondents this comparison is purely descriptive and cannot support any inferential claim; it is presented for transparency rather than as evidence that conversion does or does not influence HRQoL.

## 4 Discussion

Disease-specific HRQoL in this thoracoscopically-initiated cohort (intention-to-treat) was broadly preserved, with total QoL% of 76%–79% and no statistically significant deviation from published European norms after correction for multiple testing. At the same time, the data confirm that eating-related difficulties—particularly with food consistency—remain the dominant source of impairment regardless of age group. The strength of child–parent agreement (Pearson *r* = 0.83, near-zero Bland–Altman bias) was higher than the moderate correlations (*r* ≈ 0.4–0.6) typically reported for generic instruments (16, 17), which may have practical implications for the choice of informant in follow-up.

### 4.1 Comparison with published data

The decision to study a cohort defined by a single intended operative approach—thoracoscopy—rather than a mixed-technique series was deliberate. Mixed-technique cohorts, which dominate the existing literature (3, 5, 7, 8), cannot separate EA-related morbidity from approach-related morbidity. Thoracotomy carries well-documented late sequelae—musculoskeletal asymmetry, scoliosis, cosmetic dissatisfaction—that may independently impair body image and physical function (6). It should be acknowledged that seven patients (14%) ultimately required conversion to open thoracotomy and were retained in the cohort under an intention-to-treat principle; their HRQoL scores, where available, did not differ materially from those completed thoracoscopically (Section 3.6), although the small number of converted respondents precludes firm conclusions. In the present cohort, the impairment pattern was dominated by eating rather than body or social concerns. Whether this reflects the absence of thoracotomy-related problems or simply the natural history of EA cannot be answered without a concurrent open-repair comparator, but the pattern is at least consistent with the expected benefits of a less invasive approach.

Our overall QoL% of 76%–79% falls within the range reported in the broader literature. The fact that no domain or total score differed significantly from Swedish–German norms after Bonferroni correction (Table 3) is reassuring, though it should not be over-interpreted given the modest statistical power. The medium-sized effect sizes observed for several domains (*d* = 0.5–0.6 for Eating and Health & Treatment in the 8–17 year groups) suggest that clinically relevant differences might emerge in larger samples.

### 4.2 Eating domain

The prominence of eating impairment—particularly item P04 (food consistency)—aligns with the broader EA literature identifying dysphagia as the key long-term burden (18). Dellenmark-Blom et al. (15) showed in a larger cohort that digestive symptom burden, rather than surgical factors, drives HRQoL impairment in EA, and that a history of oesophageal dilatation was associated with worse eating scores. The modest inverse correlation between dilatation count and HRQoL we observed (*ρ* = −0.16) points in the same direction, though it fell well short of significance and should be seen as a lead for future work rather than a finding in its own right.

The GER situation in our data deserves comment. Only 13 of 51 patients had reflux documented in the operative database (25%), a figure that almost certainly understates the true burden—virtually all children born with EA experience some degree of GER (18, 19). It was precisely this anticipated under-documentation that prompted us to distribute a dedicated symptom-based reflux questionnaire to the families; because the cohort is geographically dispersed across the country, with follow-up shared between regional centres, complete prospective reflux data were difficult to retrieve from a single institutional record, and the targeted survey was the most reliable way to capture current reflux status.

Reflux was reported by 13 of 24 respondents in the questionnaire (54%), with endoscopic confirmation in 10 and pharmacological treatment (mainly proton-pump inhibitors) in 12, and a single child managed surgically by Nissen fundoplication.

This gap almost certainly reflects under-documentation in retrospective records rather than a genuine doubling of prevalence among respondents. Because reflux assessment was not fully standardised—there was no pH-impedance testing, no formally validated reflux questionnaire, and our symptom inventory was developed in-house—the extent to which GER drives eating-domain impairment in this cohort remains uncertain. Future studies would benefit from prospective integration of objective reflux data alongside EA-QOL assessment. Current ESPGHAN-NASPGHAN guidelines already recommend structured nutritional surveillance in EA patients, with attention to food texture tolerance (18). The eating-domain scores reported here are broadly in line with these guidelines.

### 4.3 Social functioning and body perception

Social functioning was well preserved (QoL%: 88%–93%), consistent with previous reports. The borderline Body and Scar finding in older children (*p* = 0.049, *d* = 0.56) did not survive correction for multiple testing and requires confirmation in larger samples. If real, it would be an unexpected result in a thoracoscopic cohort where surgical scars are typically small and inconspicuous. One possibility is that body image concerns in this age group reflect broader adolescent self-consciousness rather than scar-specific distress, but this is speculative.

### 4.4 Agreement between child and parent reports

The Pearson r of 0.83 is notably higher than what is typically reported for generic HRQoL instruments in paediatric populations, where parent–child correlations tend to cluster around 0.4–0.6 (16, 17). We suspect this reflects the concrete, behaviour-anchored nature of many EA-QOL items—food refusal, gagging, difficulty swallowing—which are directly observable by parents. With only 12 matched pairs, however, this finding is preliminary and should be replicated before drawing firm conclusions.

### 4.5 Clinical correlates

The absence of significant clinical correlates in our data mirrors findings from other small EA series (13, 15) and from larger studies where digestive symptom burden—rather than discrete surgical events—emerged as the main driver of HRQoL impairment (15). One interpretation is that HRQoL in EA is shaped primarily by the intrinsic biology of the reconstructed oesophagus (dysmotility, mucosal sensitivity, reflux burden) rather than by measurable perioperative variables. Another, equally plausible, is that our sample simply lacked the power to detect moderate associations. Prospective studies with larger samples, standardised dysphagia assessment, and objective pH-impedance data will be needed to untangle these possibilities.

Only one previous Polish study has examined HRQoL after EA repair: Rozensztrauch et al. (13) used the generic PedsQL 4.0 in a mixed Wrocław cohort and reported similarly preserved overall scores with no clinical predictors. Direct comparison of PedsQL and EA-QOL scores is not meaningful, as the two instruments measure fundamentally different constructs. What the disease-specific tool adds is the ability to detect the eating–social gradient (a 20–30 percentage point gap) that generic instruments cannot resolve. Our study appears to be the first Polish report using the EA-QOL in a cohort managed with thoracoscopy as the primary intended approach.

### 4.6 Study limitations

The sample (*n* = 25) is small, though not unusually so for a single-centre EA study—the condition is rare, and our respondent pool is comparable to most published series (3, 7, 20). The 56% response rate introduces potential selection bias. To evaluate this, we compared clinical characteristics of respondents and non-respondents: stricture rate (68% vs. 58%), conversion rate (12% vs. 15%), and median number of dilatations were similar between groups. The higher anomaly rate among non-respondents (77% vs. 56%) likely reflects the inclusion of perioperative deaths and very young ineligible children in that group rather than a systematic tendency for healthier families to respond. These comparisons provide some reassurance, although we cannot exclude the possibility that families who chose not to respond differed in ways we did not capture—for instance in terms of perceived disease severity or willingness to engage with follow-up research, neither of which was recorded.

The absence of an open-repair comparator is a significant limitation. Any claims about the relative HRQoL benefit of thoracoscopy would require a prospective study with both arms using the same instrument. Without a comparator arm, these data cannot address whether the approach itself influences outcomes. Comparison with Swedish–German reference data should be interpreted with caution. These represent an external contextual benchmark, not a validated norm for the Polish population. Cultural differences in self-report tendencies and health expectations may independently influence scores, and formal measurement invariance testing was not performed across populations.

The cross-sectional design precludes assessment of HRQoL trajectories over time—a missed opportunity in a cohort spanning nearly two decades of operations. Longitudinal follow-up would allow identification of critical windows where intervention might be most beneficial. The lack of fully standardised, objective reflux and dysphagia assessment (no pH-impedance monitoring, and reflux captured by an in-house symptom questionnaire supported by endoscopy rather than a formally validated instrument) limits interpretation of the eating domain. Finally, the entire cohort had Gross type C EA, so findings should not be extrapolated to long-gap or other anatomical variants (21, 22).

## 5 Conclusions

In this single-centre cohort managed with thoracoscopy as the primary intended approach (with a 14% conversion rate to open thoracotomy), disease-specific HRQoL was generally well maintained, with scores comparable to published European reference data. Eating difficulties—particularly problems with food consistency—were the dominant area of impairment and warrant routine monitoring through follow-up. The discrepancy between clinical-record GER documentation (25%) and prospective questionnaire-based reporting (54%) highlights the need for standardised reflux assessment in this population. Child–parent agreement was strong in this preliminary sample, supporting the use of both self-report and proxy formats in clinical practice. A prospective comparative study with an open thoracotomy arm, using the same EA-QOL instrument, would be needed to determine whether operative technique itself influences long-term HRQoL.

## Data Availability

The original contributions presented in the study are included in the article/Supplementary Material, further inquiries can be directed to the corresponding author.
