# Supplementary material for: Disease-specific quality of life after thoracoscopic repair of esophageal atresia: a single-centre cross-sectional study
Source: Front Pediatr. 2026 Jul 1;14:1844923. doi: 10.3389/fped.2026.1844923 (PMC13369439; doi:10.3389/fped.2026.1844923)
Supplement: Supplementary file 1 [file Table1.docx]

**Supplementary File 1**

*STROBE Statement — Checklist of items that should be included in reports of cross-sectional studies*

**Article:** Disease-specific quality of life after thoracoscopic repair of esophageal atresia: a single-centre cross-sectional study

| **Item No** | **Section / Topic** | **Recommendation** | **Reported on page / section** |
| --- | --- | --- | --- |
| ***Title and abstract*** | | | |
| 1a | **Title** | Indicate the study’s design with a commonly used term in the title or the abstract | Title (“cross-sectional study”) |
| 1b | **Abstract** | Provide in the abstract an informative and balanced summary of what was done and what was found | Abstract |
| ***Introduction*** | | | |
| 2 | **Background / rationale** | Explain the scientific background and rationale for the investigation being reported | Section 1, ¶1–4 |
| 3 | **Objectives** | State specific objectives, including any prespecified hypotheses | Section 1, last ¶ |
| ***Methods*** | | | |
| 4 | **Study design** | Present key elements of study design early in the paper | Section 2.1, ¶1 |
| 5 | **Setting** | Describe the setting, locations, and relevant dates, including periods of recruitment, exposure, follow-up, and data collection | Section 2.1 (Gdańsk; 2006–2025) |
| 6 | **Participants** | Give the eligibility criteria, and the sources and methods of selection of participants | Section 2.1 (N=51 eligible; 6 excluded; 45 distributed; 25 returned) |
| 7 | **Variables** | Clearly define all outcomes, exposures, predictors, potential confounders, and effect modifiers | Sections 2.2–2.3 |
| 8 | **Data sources / measurement** | For each variable of interest, give sources of data and details of methods of assessment | Sections 2.2–2.3 (EA-QOL; medical records; questionnaire GER) |
| 9 | **Bias** | Describe any efforts to address potential sources of bias | Sections 3.1, 4.6 (respondent vs. non-respondent comparison) |
| 10 | **Study size** | Explain how the study size was arrived at | Section 2.4 (no power calculation; all eligible contacted) |
| 11 | **Quantitative variables** | Explain how quantitative variables were handled in the analyses | Section 2.2 (QoL% transformation); Section 2.4 |
| 12a | **Statistical methods** | Describe all statistical methods, including those used to control for confounding | Section 2.4 (Welch’s t, Mann–Whitney, Spearman, Bonferroni, Cohen’s d) |
| 12b |  | Describe any methods used to examine subgroups and interactions | Section 2.4 (age-group stratification by questionnaire version) |
| 12c |  | Explain how missing data were addressed | Section 3.1 (1 missing GER noted) |
| 12d |  | If applicable, describe analytical methods taking account of sampling strategy | N/A |
| 12e |  | Describe any sensitivity analyses | N/A |
| ***Results*** | | | |
| 13a | **Participants** | Report numbers of individuals at each stage of study | Section 2.1 (51→6 excluded→45 distributed→25 returned) |
| 13b |  | Give reasons for non-participation at each stage | Section 2.1 (2 deaths; 4 too young) |
| 13c |  | Consider use of a flow diagram | Not used; numbers given in text |
| 14a | **Descriptive data** | Give characteristics of study participants and information on exposures and potential confounders | Table 1; Section 3.1 |
| 14b |  | Indicate number of participants with missing data for each variable of interest | Section 3.1 (1 missing GER) |
| 15 | **Outcome data** | Report numbers of outcome events or summary measures | Table 2 (means ± SD, QoL%); Table 3 |
| 16a | **Main results** | Give unadjusted estimates and, if applicable, confounder-adjusted estimates and their precision | Sections 3.4–3.6; Tables 3–4 (p-values, Cohen’s d, 95% CI for bias) |
| 16b |  | Report category boundaries when continuous variables were categorized | N/A |
| 16c |  | If relevant, consider translating estimates of relative risk into absolute risk | N/A |
| 17 | **Other analyses** | Report other analyses done — e.g., analyses of subgroups and interactions, and sensitivity analyses | Section 3.3 (item-level); Section 3.5 (Bland–Altman); Section 3.6 (bivariate correlates) |
| ***Discussion*** | | | |
| 18 | **Key results** | Summarise key results with reference to study objectives | Section 4, opening ¶ |
| 19 | **Limitations** | Discuss limitations of the study, taking into account sources of potential bias or imprecision | Section 4.6 |
| 20 | **Interpretation** | Give a cautious overall interpretation considering objectives, limitations, multiplicity of analyses, results from similar studies | Sections 4.1–4.5 |
| 21 | **Generalisability** | Discuss the generalisability (external validity) of the study results | Section 4.6 (Gross C only; no Polish norms; single centre) |
| ***Other information*** | | | |
| 22 | **Funding** | Give the source of funding and the role of the funders for the present study | Funding section |

*Reference: von Elm E, Altman DG, Egger M, Pocock SJ, Gøtzsche PC, Vandenbroucke JP; STROBE Initiative. The Strengthening the Reporting of Observational Studies in Epidemiology (STROBE) statement: guidelines for reporting observational studies. Lancet (2007) 370(9596):1453–57. doi: 10.1016/S0140-6736(07)61602-X*
